# Circadian gene CSNK1D promoted the progression of hepatocellular carcinoma by activating Wnt/β-catenin pathway via stabilizing Dishevelled Segment Polarity Protein 3

**DOI:** 10.1186/s12575-022-00183-x

**Published:** 2022-12-02

**Authors:** Mengqi Zhu, Jianping Zhang, Saiyan Bian, Xue Zhang, Yiping Shen, Zhiyu Ni, Shiyu Xu, Chun Cheng, Wenjie Zheng

**Affiliations:** 1 Research Center of Clinical Medicine, Affiliated Hospital of Nantong University, Medical School of Nantong University, Nantong, 226001 China; 2 Department of Oncology, Medical School of Nantong University, Affiliated Hospital of Nantong University, Nantong, 226001 China; 3 The First People’s Hospital of Xuzhou, Xuzhou, 221000 China

**Keywords:** Hepatocellular carcinoma, CSNK1D, Wnt, β-catenin, DVL3, Molecular target

## Abstract

**Purpose:**

A variety of studies have connected circadian rhythm to the initiation and progression of hepatocellular carcinoma (HCC). The purpose of this study was to figure out about the circadian genes' profile characteristics, prognostic significance, and targeted values in HCC.

**Methods:**

The expression profiles and prognostic significance of circadian genes in the cancer genome atlas liver hepatocellular carcinoma (TCGA-LIHC) database were investigated using bioinformatics analysis. The expression features of Casein Kinase 1 Delta (CSNK1D), a robust signature gene, was further detected by immunohistochemistry, western blotting and Real-time quantitative PCR (RT-qPCR) in a local HCC cohort. The effect of CSNK1D on corresponding phenotypes of HCC cells was evaluated using Cell Counting Kit-8 (CCK8), flowcytometry, clone assay, Transwell assay, and xenograft assay. In addition, the underlying mechanisms of CSNK1D in the Wnt/β-catenin signaling were validated by multiple molecular experiments.

**Results:**

Abnormal expression of the Circadian genome was associated with the malignant clinicopathological characteristics of HCC patients. A 10 circadian gene-based signature with substantial prognostic significance was developed using Cox regression and least absolute shrinkage and selection operator (LASSO) analysis. Of them, CSNK1D, significantly elevated in a local HCC cohort, was chosen for further investigation. Silencing or overexpression of CSNK1D significantly reduced or increased proliferation, invasion, sorafenib resistance, xenograft development, and epithelial-mesenchymal transformation (EMT) of HCC cells, respectively. Mechanically, CSNK1D exacerbated the aggressiveness of HCC cells by activating Wnt/β-catenin signaling through interacting with Dishevelled Segment Polarity Protein 3 (DVL3).

**Conclusions:**

The Circadian gene CSNK1D was found to contribute to HCC progression by boosting the Wnt/β-catenin pathway, hinting that it could be a prospective therapeutic target for HCC.

**Supplementary Information:**

The online version contains supplementary material available at 10.1186/s12575-022-00183-x.

## Introduction

Liver cancer is the sixth most prevalent malignancy and the fourth leading cause of cancer-related death worldwide. The global incidence of liver cancer is increasing, with more than 1 million people expected to be diagnosed each year by 2025 [1]. The most common type of primary liver cancer is hepatocellular carcinoma (HCC), which accounts for 75–80% of instances [2]. Surgery, along with some traditional treatments like radiofrequency ablation and transcatheter chemoembolization, is the best option for those individuals with early-stage liver cancer [3]. Furthermore, systematic therapy with targeted drugs and immune checkpoint inhibitors has been shown to improve outcomes in patients with advanced HCC [4]. Despite recent medical advances, HCC sufferers' therapeutic efficacy remains poor, with a 5-year survival rate of less than 30% [5, 6]. By decades, a range of genes and pathways have been implicated in HCC [7–9]. However, the molecular mechanisms underlying the occurrence and progression of HCC remain largely unresolved.

Circadian rhythm is generated by the negative feedback loop of the core circadian gene through transcription and translation [10]. Multiple biological functions are mediated by this rhythm, including cell cycle, apoptosis, metabolic control, and DNA damage repair [11]. The disruption of circadian rhythm is observed in most of cancer types [12], especially for prostate cancer, breast cancer, colorectal cancer, liver cancer, and lung cancer [13–15]. Furthermore, disruption of circadian rhythm modulation has been associated with poor anticancer therapeutic efficiency as well as aggressive clinicopathological features, implicating a key role in tumor occurrence and progression [16, 17]. The epidemiological evidence from animal studies suggests that some hormone-dependent cancers are particularly susceptible to circadian disruption, this may be due to the potential role of melatonin as a regulator of cell proliferation [18].

Casein kinase 1 (CK1) was sequenced and identified as 1284 nucleotide sequences, which were translated into a 49 KDa protein with 428 amino acids (Aa) [19, 20]. It's widely acknowledged as one of the most significant genes in biological rhythm regulation [10]. The circadian clock was lengthened with CK1 deficiency or inactivation, whereas overexpression of CK1 led to a shortening of the circadian rhythm [19]. CSNK1D belongs to the CK1 family, which contains seven genes encoding mammalian CK1 subtypes. CK1 has been implicated in autophosphorylation and/or phosphorylation by other cellular protein kinases, as well as interaction with cellular proteins and subcellular sequestration [21]. It has been demonstrated that CK1δ phosphorylated p53 and its negative regulator MDM2. Under normal conditions, CK1δ-mediated phosphorylation of MDM2 stabilized the MDM2-p53 complex, subsequently leading to p53 degradation [22, 23]. In addition, CK1δ/ε is critical for cell cycle progression and genomic stability. Following the administration of CK1δ/ε specific inhibitor IC261, the cells fell into a transient mitotic halt [24]. CSNK1D has been involved in multiple malignancies, including breast cancer, lung cancer, colorectal cancer, and glioblastoma [25–27]. RNA interference or pharmacological reduction of CK1δ might slow tumor growth [25, 28]. These data suggest that CK1 might be a molecular target for cancer therapy. Though High CSNK1D expression has been linked to a poor prognosis in HCC, the specific mechanism has yet to be elucidated [29].

In this study, we employed bioinformatics to examine the features of circadian rhythm genes and established a robust risk model for HCC. CSNK1D, one of the signature gene, was further evaluated for its expression profile and clinical implications. In addition, the effects of CSNK1D on the HCC phenotypes and underlying mechanisms were investigated in vitro and in vivo. This study might shed light on the roles of circadian rhythm gene in HCC progression and provide novel therapeutic targets.

## Materials and methods

### Data acquisition

The circadian rhythm genes were downloaded from Update to the integrated cancer data analysis platform (UALCAN) database (http://ualcan.path.uab.edu/). The expression profiles of the circadian rhythm genes in 374 HCC cases with clinical information were retrieved from the cancer genome atlas liver hepatocellular carcinoma (TCGA-LIHC) datasets (https://cancergenome.nih.gov/) using the R package “TCGA-Assembler”. A heatmap was used to visualize the expression levels of these genes in HCC and normal tissues. The Pearson correlation analysis was employed to evaluate the relationship between these rhythm genes.

### Construction of prognostic signature

A univariate Cox regression model was used to assess the relationship between 28 circadian rhythm genes and the overall survival of HCC patients. A risky gene was defined as one with hazard ratio (HR) more than 1, whereas one with HR less than 1 was considered protective. 14 genes risk genes (CSNK1D, TIPIN, NPAS2, GSK3B, RORC, CRY2, CSNK1E, PER1, ARNTL2, SERPINE1, TIMELESS, NR1D1, RORA, SENP3) were correlated with overall survival. To establish the risk signature, 10 genes were chosen based on the minimal criterion. The risk score was calculated using the expression value and the coefficients in the least absolute shrinkage and selection operator (LASSO) algorithm. Based on the median value of the risk scores, the TCGA-LIHC cohort was divided into high-risk group and low-risk group. The distribution of clinicopathologic features (age, gender, grade stage, and survival state) in high- and low-risk groups was further evaluated using the chi-square test and shown with heatmaps.

### Evaluating the prognostic value of the gene signature

A receiver operating characteristic (ROC) curve was used to assess the signature's predictive efficacy in predicting survival outcomes. A Kaplan–Meier analysis with a log-rank test was used to examine the difference of overall survival between patients in the high- and low-risk groups. The risk score was then evaluated as an independent prognosis predictor for HCC patients in the TCGA cohort using Univariate and Multivariate Cox regression analysis. In addition, the risk model was investigated in subgroups depending on stage and grade level.

### Expression profiles and functional prediction for CSNK1D

The mRNA profiles of CSNK1D in Pan-cancers was analyzed by TCGA database (https://cancergenome.nih.gov/). Microarray data of Gene Expression Omnibus Series (GSE)19665 and GSE62232 was downloaded from the National Center for Biotechnology Information (NCBI) Gene Expression Omnibus (GEO, http://www.ncbi.nlm.nih.gov/geo/) database. The gene expression value was calculated using the average expression value of multiple probes belonging to one gene. The preprocess Core package (version 1.28.0, http://www.bioconductor.org/ packages/release/bioc/html/preprocessCore.html) was then performed to normalize gene expression values using the log2 transformation. To identify enriched functions and pathways associated with CSNK1D, gene set enrichment analysis (GSEA) was performed on the high- and low-subgroups of the TCGA cohort. In this study, GSEA v.3.0 and the Molecular Signatures Database v.7.0 were employed. Significantly enriched gene sets were defined as those with a P value less than 0.05 and a False discovery rate (FDR) less than 25%.

### Clinical samples of local cohort

Tissue samples of the Nantong cohort were collected from 110 HCC patients who underwent surgery at the Affiliated Hospital of Nantong University (Nantong, Jiangsu, China) between January 2012 and October 2014. The study was conducted in compliance with the Helsinki Declaration and was approved by the ethical committee of Nantong University Affiliated Hospital.

### Cell culture and transfection

HCC cell lines HepG2 (catalog number, #SCSP-510), Hep3B (catalog number, #SCSP-5045), and BEL-7404 (catalog number, #TCHu64) were provided by the Cell Bank of the Chinese Academy of Sciences (Shanghai, China). MHCC97H (catalog number, #ZQ0020), and HCCLM3 (catalog number, #ZQ0023) were provided by Zhong Qiao Xin Zhou Biotechnology (Shanghai, China). Dulbecco's modified Eagle's medium (DMEM; Gibco, USA) was used to culture the cells, supplemented with 10% fetal bovine serum (FBS; Gibco, USA) and 1% penicillin/streptomycin solution. Plasmids coding for CSNK1D (pTSB-CMV-CSNK1D-copGFP-F2A-PuroR) were designed and purchased from TransheepBio-Tech CO, LTD (Shanghai, China). To overexpress CSNK1D, HepG2 cells were transfected with OE-CSNK1D plasmids or control vector plasmids by Lipofectamine 3000(Invitrogen). Forty-eight hours later, 5 µg/ml of puromycin (Invitrogen) was added to the medium and maintained for another 14 days until the stably-transfected cell lines were established. The sequence of short hairpin RNA(ShRNA) with highest intervening efficacy targeting CSNK1D was CUAUCUCGGUACGGACAUUTTAAUGUCCGUACCGAGAUAGTT. The sequence of small interfering RNA (siRNA) with highest intervening efficacy targeting Dishevelled Segment Polarity Protein 3 (DVL3) was GCUCCCUUUCACCAUUUAUAUAAAUGGUGAAAGGGAGC.

### Cell counting kit‑8, colony formation assays and tumor sphere formation

Following CSNK1D knockdown or overexpression, cell proliferation was measured using the Cell Counting Kit-8 assay (CCK-8; Dojindo Laboratories, Kumamoto, Japan) according to the manufacturer's instructions. For colony formation evaluation, HCC cells of each group were cultured in the six-well plate at a density of 100/well. After 14 days of incubation, the samples were fixed in 4% paraformaldehyde (PFA) for 30 min and stained with 0.1 percent crystal violet solution. Ultra-low adhesion 6-well plates were utilized for tumor sphere formation, and 5000 cells were plated per well, supplemented with 2 mL StemXVivo serum-free tumor sphere medium (CCM012, R&D Systems, Minneapolis, MN, USA), 2 U/mL heparin (Sigma, CA, USA), and 0.5 g/mL hydrocortisone (Sigma, CA, USA). Cells were cultured for 7–10 days at 37 °C in 5% CO2. Tumor spheres were then counted and images were obtained at a magnification of 40.

### Wound healing and transwell assay

The wound-healing assay was performed for migration evaluation. Cells were cultivated in the 6-well plate for 24 h, and subsequently mechanically scratched by a sterile serum tube. Scratch healing were measured at different time points. The invasion assay was conducted using 8-μm Transwell chambers (Corning, Acton, MA, USA). 200 μL suspension of MHCC97H or HepG2 cells were plated in the upper chambers, while the complete medium was plated in the lower chamber. After 48 h, the chambers were fixed for 40 min at room temperature with 4% paraformaldehyde, stained with crystal violet (C0121, Beyotime), and counted with ImageJ.

### Cell cycle and apoptosis analysis

The effects of CSNK1D on HCC ell apoptosis were investigated using a FITC-Annexin V Apoptosis Detection Kit (BD Biosciences, USA) in accordance with the manufacturer's instructions. Each group's cells were pre-suspended in binding buffer before being treated with Annexin V-FITC for 15 min. The samples were analyzed using a BD FACS Calibur flow cytometer (Becton–Dickinson, USA) after incubation with propidium iodide (PI). For cell cycle analysis, cells from each group were treated with PI solution for 15 min before being detected using a BD FACS Calibur flow cytometer. Modfit software was used to examine the results.

### Xenograft tumor assay

Male BALB/c nude mice were obtained from Nantong University's Laboratory Animal Center. The subcutaneous injection paradigm was employed to examine HCC cell growth in vivo. 3 × 10^6^ CSNK1D-overexpressed HepG2 cells or control cells were suspended in 100L of PBS and subcutaneously injected into the flanks of nude mice. Tumor growth was measured every three days after injection using calipers. The volume of the xenograft tumor was determined to be 0.5 × length × width^2^. Tail vein injection was conducted to assess the metastatic ability of HCC cells in vivo. HCC cells were transplanted into nude mice by tail vein injection. At 10^th^ week after implantation, the lungs of nude mice were collected and fixed in paraffin for hematoxylin–eosin (H&E) staining. The protocols of this study were approved by the Animal Care and Use Committee of Nantong University.

### Immunohistochemistry and immunofluorescence

Deparaffinized paraffin-embedded sections were deparaffinized in xylene before being rehydrated in a graded series of ethanol. Sodium citrate buffer was used to retrieve antigens (10 mM sodium citrate, 0.05% Tween 20, pH 6.0). Slides were boiled for 10 min, and were then cooled to room temperature. Endogenous peroxidase activity was blocked by 3% hydrogen peroxide in methanol for 20 min, followed by 5 washes with tris-buffered saline (TBS) containing 0.025% Triton X-100. Then the slides were blocked using 10% normal serum with 1% BSA in TBS for 2 h at room temperature. The slides were blocked for 2 h at room temperature with 10% normal serum and 1% bovine serum albumin (BSA) in TBS. The sections were then incubated with the primary and secondary antibodies in the order listed. The primary antibodies were diluted as follows: CSNK1D (1:200, #ab85320, Abcam, USA), Ki67 (1:200, #ab16667, Abcam, USA), β-catenin (1:100, #8480, CST, USA), N-Cadherin (1:100, #A19083, ABclonal, China), E-Cadherin (1: 200, #3195, Cell Signaling Technology, USA), Vimentin (1:200, #10,366–1-AP, Proteintech, China), DVL3 (1:200, #13,444–1-AP, Proteintech, China). The secondary antibody (#A0208, #A0216, Beyotime, China) was diluted as a concentration of 1:50. Diaminobenzidine (DAB) staining was used for the final visualization. For immunofluorescence, cells were fixed and permeabilized with 4% formaldehyde and 0.25% Triton X-100 for the cell immunofluorescence assay, then blocked for 1 h at room temperature with 1% BSA. Cells were washed and incubated with Fluor-labeled secondary antibody after 12 h of incubation with the primary antibody dilution, followed by imaging using a fluorescence microscopy. The primary antibodies were diluted as follows: β-catenin (1:50, #8480, CST, USA), CSNK1D (1:50, #sc-55553, SantaCruz, USA), DVL3 (1:100, #13,444–1-AP, Proteintech, China). Red rabbit fluorescent antibody (1:100, #AS007, ABclonal, China), Red mouse fluorescent antibody (1:100, #AS008, ABclonal, China), and Green fluorescent antibody (1:100, #AS011, ABclonal, China) were used as the secondary antibody, respectively. The nuclear was visualized by 4’,6-Diamidino-2’-phenylindole (DAPI) dyeing solution (#C1005, Beyotime, China).

### Western blotting, immunoprecipitation and protein stability

Radioimmunoprecipitation assay buffer (RIPA) lysis buffer (P0013B, Beyotime) with protease inhibitors and phosphatase inhibitors (Roche) was used to lyse liver tissues and hepatoma cells. Then sodium dodecyl sulfate polyacrylamide gel electrophoresis (SDS-PAGE) sample loading buffer (Beyotime) was added to the lysate and boiled in a water bath. The protein sample was separated by SDS gels and then transferred to polyvinylidene fluoride (PVDF) Membranes with a thickness of 0.22 µm (Sigma, USA). After that, the membranes were treated with primary antibodies CSNK1D (1:1000, ab85320, Abcam), GAPDH (1:1000, #60,004–1-Ig, Proteintech), E-Cadherin (1:500, #3195, CST), N-Cadherin (1:500, #A19083, ABclonal), Vimentin (1:2000, #10,366–1-AP, Proteintech), Sanil(1:1000, #3879, CST), β-catenin (1:1000, #8480, CST), GSK-3β(1:1000, #9315, CST), p-GSK-3β(1:1000, #5558, CST), c-Myc(1:500, #sc-40, SantaCruz), cyclinD1(1:500, #2978, CST), DVL3(1:1000, #13,444–1-AP, Proteintech) at 4 ℃ overnight. The membranes were washed and incubated with corresponding secondary antibodies (1:1000, #A0208, A0216, Beyotime). NcmECL Ultra (P10300A/B, NCM Biotech) was used to detect the blotting. RIPA lysis solution (P0013C, Beyotime) containing protease inhibitors was used to lyse cells for immunoprecipitation. Isolated lysates were precleared with protein A/G agarose (Bioepitope, China), then immunoprecipitated with primary antibodies overnight, followed by a 2.5-h incubation with protein A/G agarose. The precipitates were eluted and subjected to SDS gels for immunoblotting (IB) assays. For protein stability assay, cells were administrated with CHX (100 μg/ml, Sigma, CA, USA) and collected at different time points, thereby conducting western blotting to detect the protein degradation.

### Reverse transcription quantitative polymerase chain reaction (RT‑qPCR)

Total RNA was extracted using Trizol reagent (Invitrogen, USA) according to the manufacturer’s protocols and then quantified by Nanodrop spectrophotometry. The BeyoRTTMII First Strand complementary DNA Synthesis Kit was used for reverse transcription (Beyotime). Quantitative real-time PCR was performed on LightCycler480II (Roche) system with SYBR green mix kit (Vazyme). The parameters of PCR were set as follows: Pre-denaturation at 95 °C for 5 min, then 40 cycles at 95 °C for 10 s, followed by 60 °C for 30 s. Dissolve curve at 95 °C for 15 s, 60 °C for 60 s, and 95 °C for 15 s. The glyceraldehyde phosphate dehydrogenase (GAPDH) was used as a loading control. All expression Ct values of target genes were analyzed by the 2-ΔΔCT methods. The primers used were listed as follow: CSNK1D (NM_001893): F-AAGTCACGTTGTCTCGAAGCATGG; R-TGAAGCCAAGCCGCAAGGTAAC; GAPDH (NM_001256799): F-GGAGCGAGATCCCTCCAAAAT, R-GGCTGTTGTCATACTTCTCATGG.

### Statistical analysis

The data were presented as means with standard deviations and were deemed statistically significant at *p* < 0.05. The data were analyzed using IBM SPSS Statistics 20 (IBM Corporation, USA). For group comparisons, the Student's t test was utilized. The chi-square or Fisher's exact tests were used to assess categorical data. The GraphPad Prism program (GraphPad, USA) or R software were used to evaluate group comparisons. Survival analyses were carried out using Kaplan–Meier with a log-rank testing. Statistical significance was determined as follows: *P < 0.05, **P < 0.01, ***P < 0.001, ****P < 0.0001.

## Results

### Construction of circadian genes-based signature

**Fig. 1:**
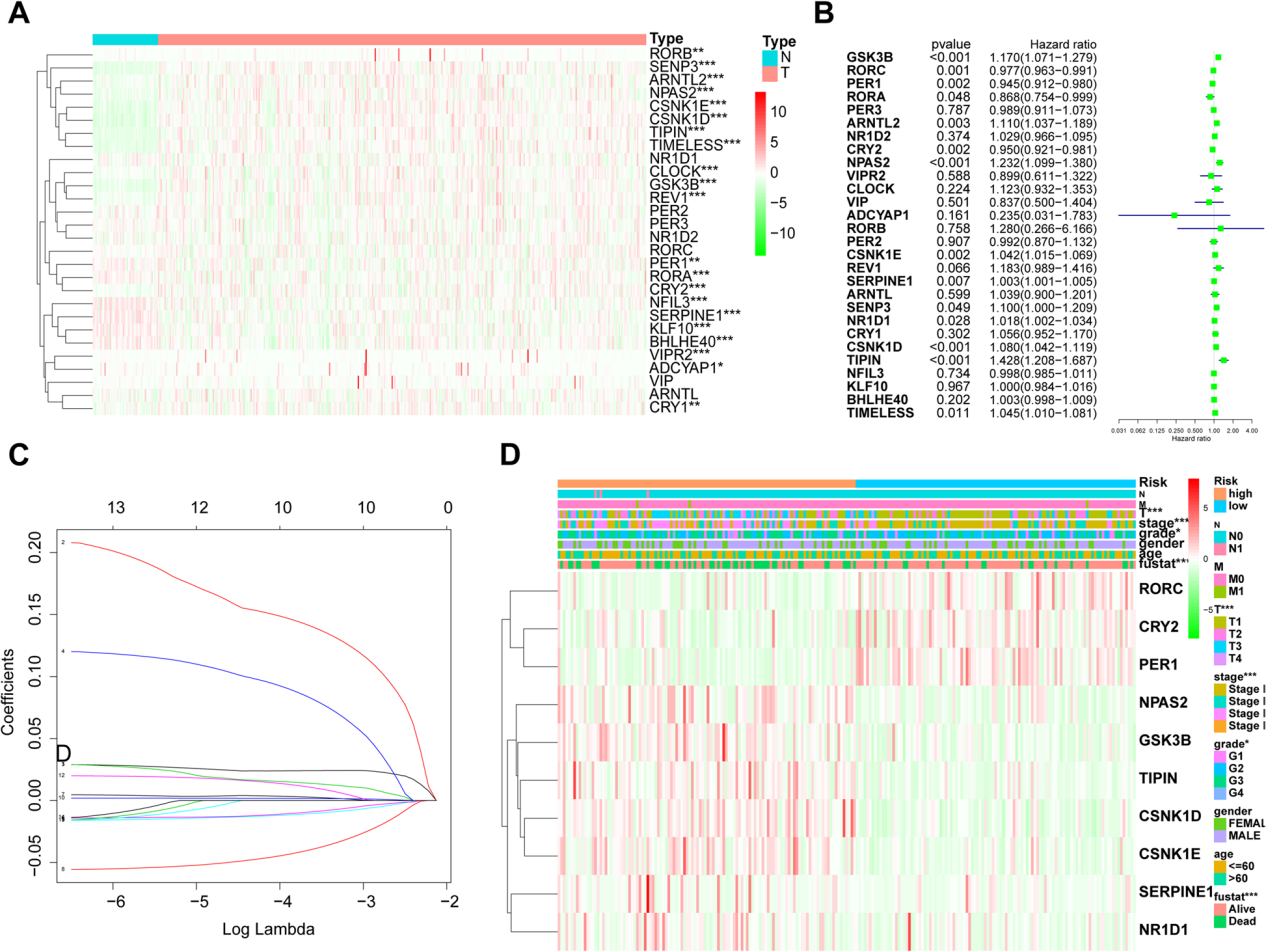
Genomic Alterations of the Circadian genes in TCGA LIHC Cohort **A** The heatmap showed the expression profiles of 28 circadian genes in HCC and normal liver tissue from the Cancer Genome Atlas (TCGA) liver hepatocellular carcinoma (LIHC) dataset. **B** Univariate Cox regression was performed to screen circadian genes with prognostic value. **C **Ten- circadian gene based signature with coefficients (CSNK1D, TIPIN, NPAS2, GSK3B, RORC, CRY2, CSNK1E, PER1, SERPINE1, NR1D1) was calculated by multivariate Cox regression with least absolute shrinkage and selection operator (LASSO). **D** Expression characteristics and clinicopathological distribution of the signature genes in TCGA LIHC datasets

Circadian gene expression profiles were examined in the TCGA LIHC cohort. When compared to normal liver tissue, most Circadian genes were overexpressed in liver cancer tissues (Fig. 1A). Then, we screened the circadian genes that were associated with the prognosis of HCC patients. Univariate Cox regression analysis indicated that 15 genes were significantly correlated with HCC prognosis (Fig. 1B). The prognostic signature was then established using multivariate Cox analysis with LASSO, a generalized linear model. Following the log2 transformation of the lambda (λ) value, which was determined by the smallest likelihood deviance, a coefficient profile plot was generated. Ten Circadian genes (CSNK1D, TIPIN, NPAS2, GSK3B, RORC, CRY2, CSNK1E, PER1, SERPINE1, and NR1D1) with coefficients were identified with minimum tenfold cross-validated mean square error in TCGA cohort (Fig. 1C). The HCC cohort was divided into high-risk and low-risk groups based on the median risk score of the circadian genes-based score. The high-risk group was associated with aggressive characteristics such as T status, tumor stage, and histological grade (Fig. 1D).

### Prognostic significance of the circadian genes-based signature

**Fig. 2:**
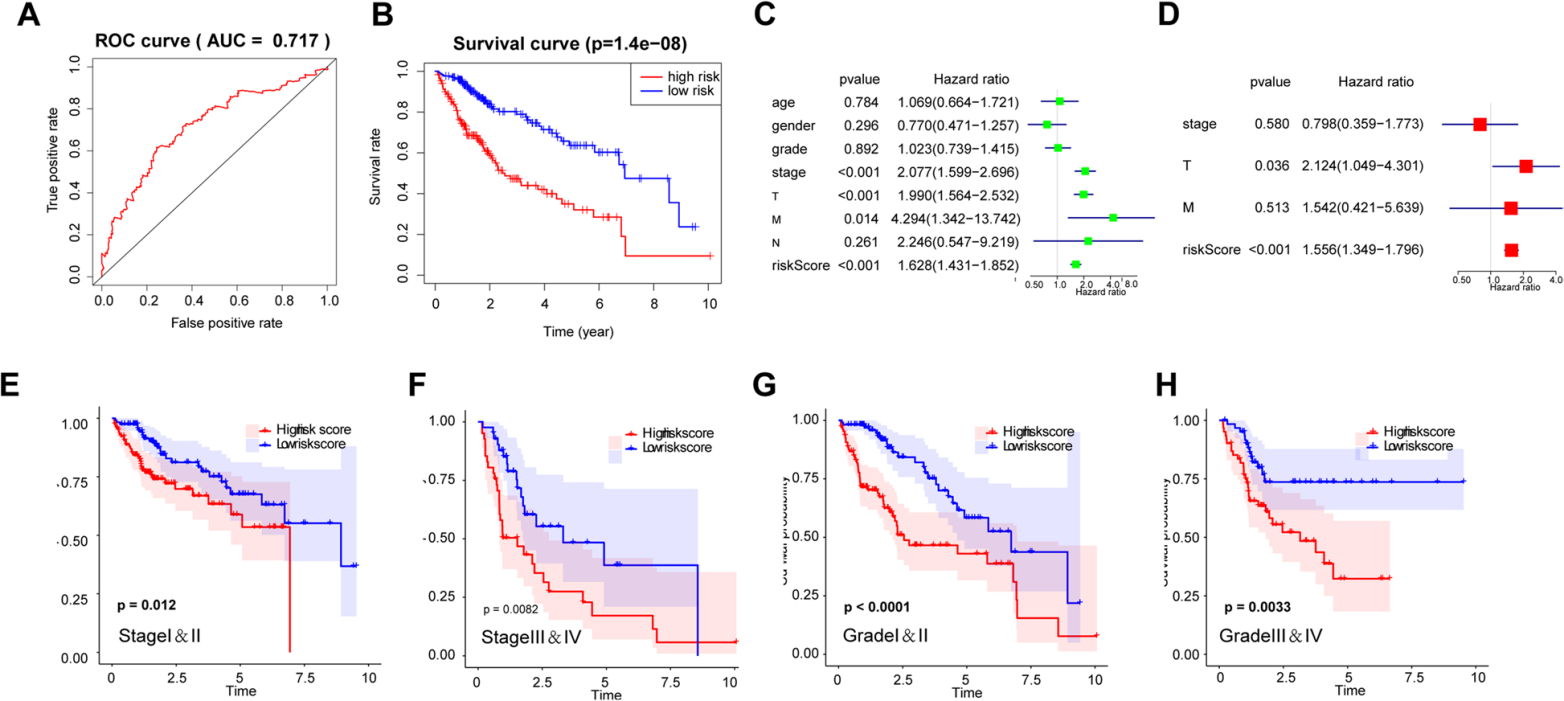
Prognostic significance of the circadian genes-based signature. **A** Receiver operating characteristic (ROC) curves were calculated to evaluate the predictive efficiency of the circadian signature. **B** The Kaplan–Meier curves of HCC patients at the high-risk score group and low-risk score group in TCGA cohort. **C, D** Univariate and Multivariate Cox analyses of the clinicopathological factors (including the risk score) and overall survival in TCGA LIHC cohort. **E, F** The Kaplan–Meier curves for HCC patients at early or advanced stages in TCGA cohort. **G, H** The Kaplan–Meier curves for HCC patients at early or advanced stages in TCGA cohort

Then we assessed the prognostic value of the circadian genes-based signature. As shown in Fig. 2A, the risk score of the signature could predict survival status for HCC patients (AUC = 0.717). In addition, Kaplan Meier analysis showed that HCC patients with high-risk scores had significantly shorter overall survival than low-risk patients (*p* < 0.001, Fig. 2B). The Univariate Cox analysis showed that the signature risk score, tumor stage, T status, M status were potential risk factors (Fig. 2C). Further multivariate Cox regression analysis revealed that risk score (*P* < 0.001, HR = 1.556, 95%CI = 1.349–1.796) and T status were independent factors for HCC prognosis (Fig. 2D). Furthermore, stratified analysis was performed to evaluate the prognostic value of the signature in subgroups. A high-risk score resulted in a poor overall survival of HCC patients in both early and advanced stages (Fig. 2E and F). Consistently, shorter OS was observed in high-risk patients at both high and low grades (Fig. 2G and H).

### The clinical implication of CSNK1D overexpression in HCC

**Fig. 3:**
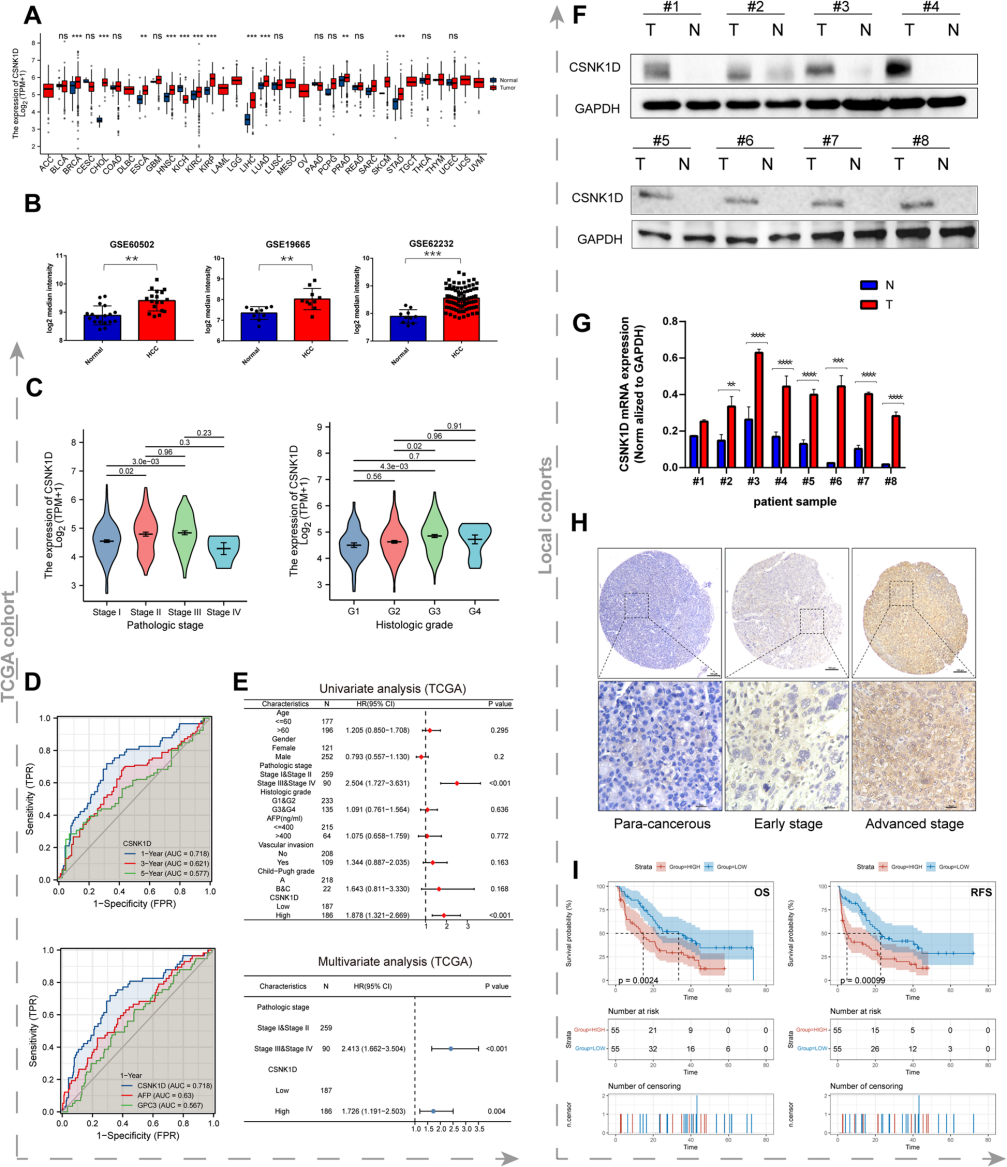
The expression features and clinical implication of CSNK1D in HCC. **A** Expression characteristics of CSNK1D in different cancer types in TCGA database. **B** The expression levels of CSNK1D in GSE19665, GSE60502 and GSE62232. **C** CSNK1D expression in HCC patients at different subgroups. **D** The ROC of CSNK1D in different time periods and comparison with typical HCC markers. **E** Univariate and Multivariate Cox analyses of the clinicopathological factors in TCGA LIHC cohort. **F, G** Analysis of CSNK1D protein and mRNA expression in 8 matched pairs of HCC and adjacent tissues. **H** The representative immunohistochemical staining of CSNK1D in adjacent tissues and HCC tissues at different stages. **I** The overall survival and recurrence free survival of HCC patients with high or low CSNK1D expression identified by immunohistochemistry. **P < 0.01, ***P < 0.001, ****P < 0.0001

Then, we focused on CSNK1D, one of the signature genes, due to its possible roles in HCC progression. First, we explored the expression features and clinical value in bioinformatic datasets. According to the observation in TCGA, CSNK1D was highly expressed in multiple tumors, in which LIHC had the most significant fold change between tumor and normal tissues (Fig. 3A). Consistently, higher CSNK1D expression of HCC tissue was also confirmed in HCC tissues from three GEO datasets (Fig. 3B). As shown in Fig. 3C, CSNK1D is overexpressed in advanced stages or grades. ROC analysis revealed that it has a high predictive value for HCC prognosis, particularly when compared to the typical HCC markers GPC3 and AFP (Fig. 3D). Meanwhile, we discovered that elevated CSNK1D expression was associated with poor overall survival (OS) and Recurrence free survival (RFS) throughout time periods (Figure S1). The univariate and multivariate analysis suggested CSNK1D was an independent marker for HCC prognosis in TCGA cohort (Fig. 3E).

Then, we further detected CNSK1D expression in 8 pairs of local HCC samples. CSNK1D expression was considerably higher in 8 HCC tissues at both protein and mRNA levels as compared to self-paired adjacent tissues (Fig. 3F&G). The expression of CSNK1D protein was then detected using immunohistochemistry in 110 cases of self-paired HCC tissues from local HCC cohort. HCC tissues, as shown in Fig. 3H, had a higher CSNK1D staining intensity than adjacent tissues, especially in advanced HCC cases. Then, we further analyzed the correlation of CSNK1D with clinical features in 110 HCC patients. High expression of CSNK1D was significantly associated with tumor size, tumor differentiation, microvascular invasion, TNM stage, metastasis and recurrence (Table 1). Then, Kaplan–Meier analysis revealed that elevated CSNK1D expression was associated with poor OS and RFS of HCC patients (Fig. 3I). Univariate and multivariate analyses consistently showed that CSNK1D was an independent predictor of HCC patients' OS and RFS (Table 2 and Table 3). Thus, CSNK1D was a robust biomarker for predicting survival of HCC patients.

**Table 1:** Correlation of CSNK1D expression with clinical features in 110 HCC patients

Parameters	n	CSNK1D expression		P
		**Low**	**High**	
**Sex**				0.065
Male	93	43	50	
Female	17	12	5	
**Age**				0.335
< 50	63	29	34	
≥ 50	47	26	21	
**AFP(ng/ml)**				0.175
< 400	65	36	29	
≥ 400	45	19	26	
**Tumor size**				0.01
< 5 cm	41	27	14	
≥ 5 cm	69	28	41	
**Differentiation**				< 0.001
Well	35	32	3	
Moderate & Poor	75	23	52	
**Microvascular Invasion**				0.021
Yes	24	7	17	
No	86	48	38	
**TNM stage**				< 0.001
I-II	39	30	9	
III-IV	71	25	46	
**Tumor number**				0.825
Single	83	41	42	
Multiple	27	14	13	
**HBV-DNA**				0.849
< 1000	55	28	27	
≥ 1000	55	27	28	
**Liver cirrhosis**				0.563
Yes	63	33	30	
No	47	22	25	
**Metastasis**				0.012
Yes	6	0	6	
No	104	55	49	
**Recurrence**				0.008
Yes	52	19	33	
No	58	36	22	

*AFP* alpha-fetoprotein, *TNM* tumor-node-metastasis

**Table 2:** Univariate and multivariate analyses of the overall survival in 110 HCC cases

Parameters	Univariate analysis			Multivariate analysis		
	**HR**	**95%CI**	**P**	**HR**	**95%CI**	**P**
**CSNK1D expression**						
High vs. low	2.016	1.268–3.204	0.003	1.937	1.217–3.084	0.005
**Sex**						
Male vs. female	0.849	0.456–1.581	0.606			
**Age**						
< 50 vs. ≥ 50	0.793	0.497–1.266	0.331			
**AFP**						
< 400 vs. ≥ 400	1.735	1.095–2.750	0.019	1.642	1.034–2.606	0.066
**Tumor size**						
< 5 vs. ≥ 5	2.477	1.480–4.147	0.001	1.745	1.029–2.959	0.059
**Tumor differentiation**						
Well vs. Moderate & Poor	2.352	1.386–3.990	0.002	1.773	0.969–3.244	< 0.001
**Microvascular invasion**						
Yes vs. No	3.224	1.905–5.456	< 0.001	2.388	1.392–4.098	0.082
**TNM stage**						
I-II vs. III-IV	2.594	1.531–4.394	< 0.001	1.855	1.040–3.309	0.036
**Tumor number**						
Single vs. Multiple	2.706	1.652–4.432	< 0.001	3.072	1.826–5.166	0.052
**HBV-DNA**						
< 1000 vs. ≥ 1000	1.325	0.838–2.097	0.229			
**Liver cirrhosis**						
Yes vs. No	1.083	0.682–1.718	0.736			
**Metastasis**						
Yes vs. No	1.434	0.521–3.949	0.485			

*AFP* alpha-fetoprotein, *TNM* tumor-node-metastasis

**Table 3:** Univariate and multivariate analyses of the recurrence-free survival in 110 HCC cases

Parameters	Univariate analysis			Multivariate analysis		
	**HR**	**95%CI**	**P**	**HR**	**95%CI**	**P**
**CSNK1D expression**						
High vs. low	2.133	1.344–3.386	0.001	1.758	1.039–2.972	0.035
**Sex**						
Male vs. female	0.958	0.516–1.778	0.892			
**Age**						
< 50 vs. ≥ 50	0.678	0.425–1.080	0.102			
**AFP**						
< 400 vs. ≥ 400	1.736	1.099–2.740	0.018	1.665	1.053–2.634	0.069
**Tumor size**						
< 5 vs. ≥ 5	2.297	1.386–3.809	0.001	1.749	1.036–2.951	0.097
**Tumor differentiation**						
Well vs. Moderate & Poor	1.954	1.168–3.268	0.011	1.885	1.125–3.157	0.003
**Microvascular invasion**						
Yes vs. No	4.696	2.681–8.226	< 0.001	4.016	2.210–7.299	0.061
**TNM stage**						
I-II vs. III-IV	2.522	1.501–4.238	< 0.001	1.887	1.089–3.271	0.024
**Tumor number**						
Single vs. Multiple	2.382	1.461–3.884	< 0.001	2.530	1.507–4.248	0.051
**HBV-DNA**						
≤ 1000 vs. > 1000	1.130	0.718–1.778	0.599			
**Liver cirrhosis**						
Yes vs. No	1.130	0.714–1.789	0.602			
**Metastasis**						
Yes vs. No	1.154	0.421–3.168	0.781			

*AFP* alpha-fetoprotein, *TNM* tumor-node-metastasis

### CSNK1D enhanced the aggressive behaviors and facilitated EMT phenotypes in HCC cells

**Fig. 4:**
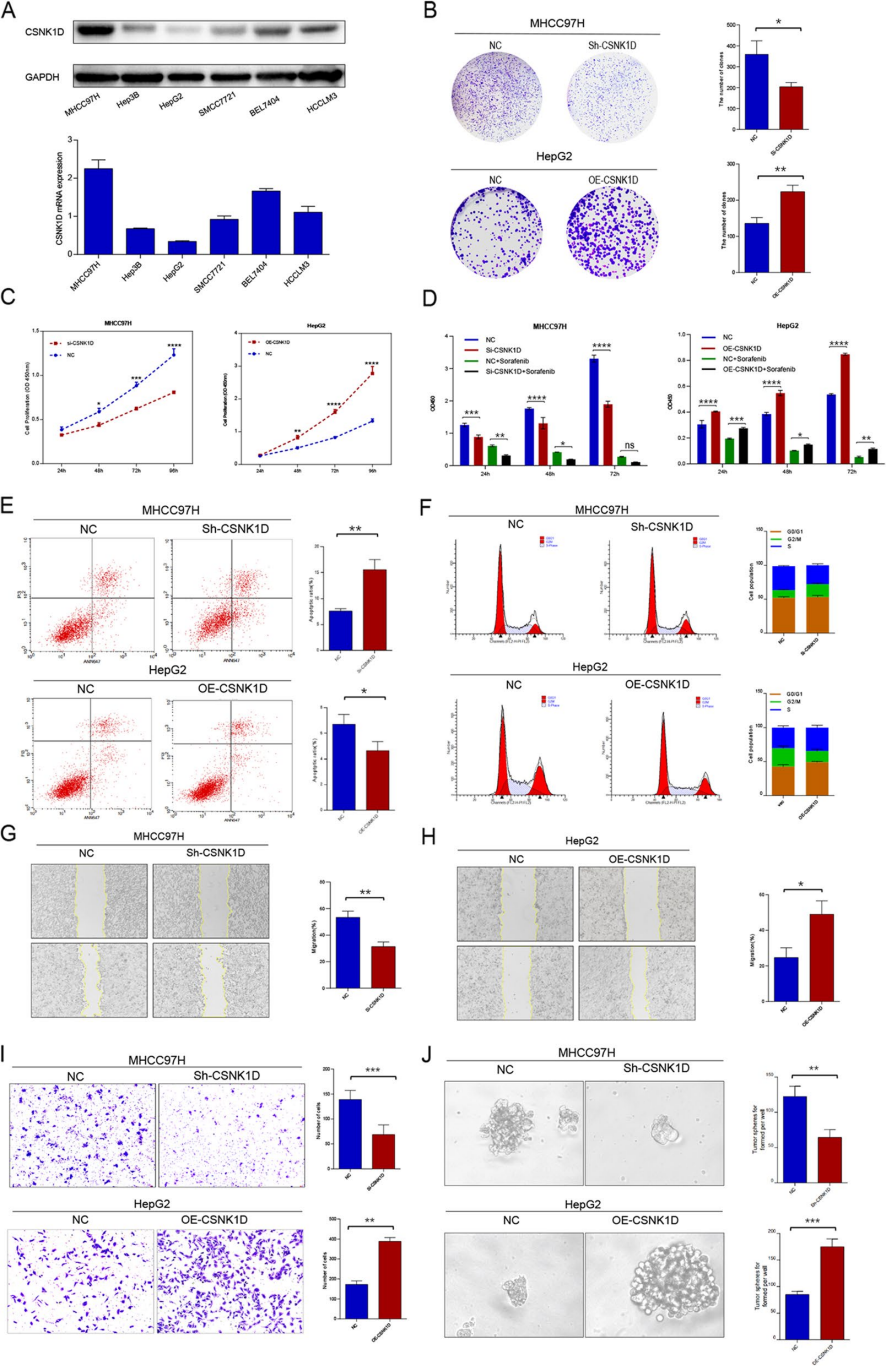
CSNK1D enhanced the aggressive behaviors and facilitated EMT phenotypes in HCC cells. **A** The protein and mRNA level of CSNK1D in HCC cell lines was detected by Western blotting and RT-qPCR. **B** Colony formation assay was conducted to assess cell survival of MHCC97H and HepG2 cells. **C** The effect of CSNK1D on proliferation of MHCC97H and HepG2 cells was evaluated by performing CCK-8 assay. **D** Sorafenib sensitivity of HCC cells with CSNK1D knockdown or CSNK1D overexpression. **E, F** The cell cycle and apoptosis were detected by flowcytometry. **G-I** The migration activity of MHCC97H and HepG2 cells was assessed by performing wound healing assay and Transwell assay. **J** Cancer stem cell property was detected by sphere formation assays. *P < 0.05, **P < 0.01, ***P < 0.001, ****P < 0.0001

We further investigated the involvement of CSNK1D in the biological behavior of HCC cells in light of its expression characteristics and clinical implications. Differential CSNK1D expression was observed in HCC cell lines at both the protein and mRNA levels (Fig. 4A). Then loss-function and gain-function assays were performed in MHCC97H cells and HepG2 cells, respectively. As shown in Fig. 4B-D, knockdown of CSNK1D significantly reduced MHCC97H cells colony formation, proliferation, and sorafenib resistance of, whereas overexpressing CSNK1D enhanced these aggressive behaviors in HepG2 cells. Furthermore, CSNK1D silencing or overexpression increased or decreased G2/M phase arrest and apoptosis of MHCC97H or HepG2 cells, respectively (Fig. 4E and Fig. 4F). Additionally, CSNK1D remarkably strengthened the migration ability of HCC cells (Fig. 4G-I). As presented in Fig. 4J, CSNK1D overexpression reinforced the sphere formation ability, while CSNK1D silencing downregulated the cancer stem cell-like property. Therefore, CSNK1D might act as a modulator of HCC cells' aggressive behaviors.

### CSNK1D activated the Wnt/β-catenin signaling of HCC cells

**Fig. 5:**
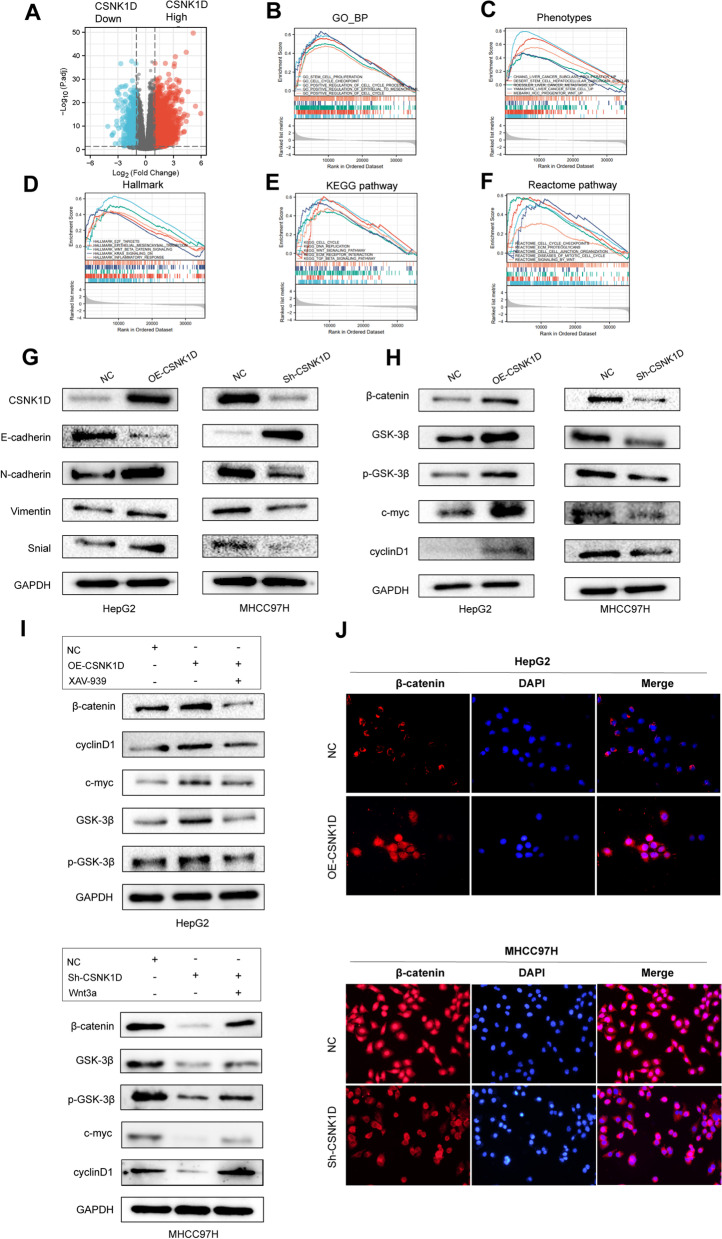
CSNK1D activated the Wnt/β-catenin signaling of HCC cells. **A **The volcano figure identifying the DEGs between CSNK1D-high and CSNK1D-low patients in TCGA dataset. **B-F** Gene sets enrichment analysis (GSEA) of the CSNK1D-mediated pathways in TCGA LIHC cohort. **G** The EMT markers were detected by Western blotting. **H** The markers of Wnt/β-catenin signaling were detected by Western blotting. **I** The activity of Wnt/β-catenin pathway was detected with CSNK1D overexpression and XAV939 treatment, along with CSNK1D knockdown and Wnt3a treatment. **J** The expression and location of β-catenin was detected by immunofluorescence in HepG2 and MHCC97H cells with CSNK1D overexpression and knockdown, respectively

We utilized GSEA-based prediction to delve deeper into the underlying mechanisms. Differential expressed genes (DEGs) between CSNK1D-high and CSNK1D-low patients in TCGA LIHC cohort were identified with the threshold of |log2(FC)|> 1 and p.adj < 0.05 (Fig. 5A). Subsequently, GSEA indicated that CSNK1D was implicated in a variety of HCC phenotypes, including cell cycle, epithelial-mesenchymal transition (EMT), liver cancer stem cell (CSC), liver cancer metastasis and HCC progenitor (Fig. 5B and Fig. 5C). As illustrated in Fig. 5D-F, CSNK1D might be correlated with the activity of the Wnt/β-catenin signaling pathway, which is identified as a typical metastasis and proliferation-related pathway [30]. Consistently, experimental assays confirmed that CSNK1D promoted EMT of HCC cells, as evidenced by increased N-cadherin, Vimentin, and Snail expression and decreased E-cadherin expression (Fig. 5G). We then examined whether CSNK1D could modulate the canonical Wnt/β-catenin pathway. Deletion of CSNK1D in MHCC97H cells reduced the expression of β-catenin, GSK-3β, P-GSK-3β, c-Myc and cyclinD1, whereas ectopic CSNK1D elevated the expression of these markers in HepG2 cells (Fig. 5H). XAV-939, a Wnt pathway inhibitor, greatly reduced the activation of the Wnt/β-catenin pathway caused by CSNK1D overexpression. In contrast, Wnt3a rescued the activity of Wnt/β-catenin pathway repressed by CSNK1D knockdown (Fig. 5I). Furthermore, immunofluorescence revealed that overexpression or knockdown of CSNK1D increased or decreased β-catenin expression with affecting nuclear translocation, which is a hallmark of Wnt/β-catenin signaling activation (Fig. 5J).

### CSNK1D activates the Wnt/β-catenin signaling pathway by interacting with DVL3

**Fig. 6:**
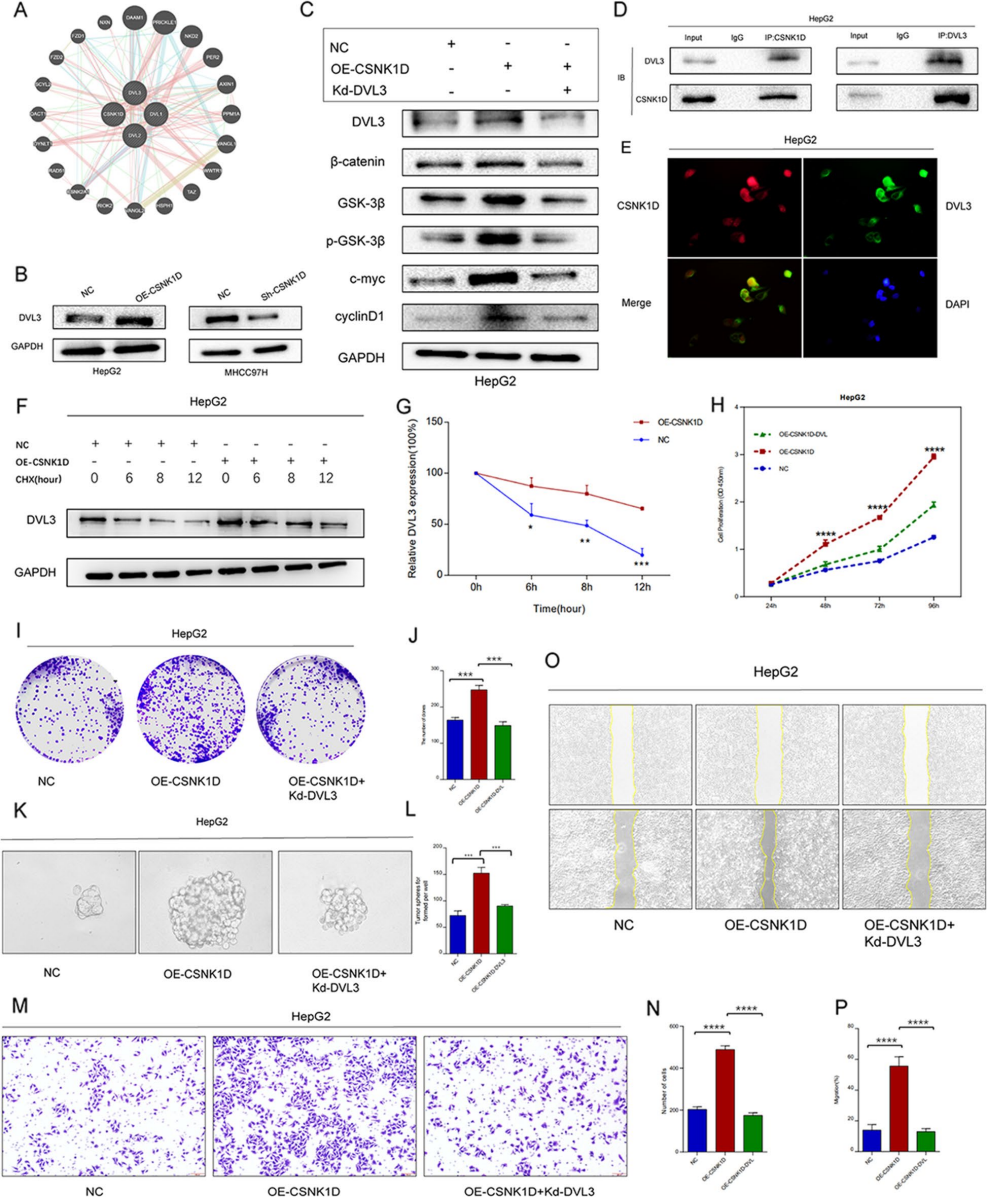
CSNK1D activates the Wnt/β-catenin signaling pathway by interacting with DVL3. **A** CSNK1D interactors were predicted by GENEMANIA database. **B** DVL3 expression was detected with silencing or overexpressing CSNK1D. **(C)** Key proteins of Wnt/β-catenin pathway was detected with CSNK1D overexpression and DVL3 silencing. **D** The interaction between CSNK1D and DVL3 were determined by Co-IP assay in CSNK1D-overexpressed HepG2 cells. **E** CSNK1D and DVL3 were mainly co-localized in cell nucleus as determined by immunofluorescence. **F, G** The half-life of DVL3 protein in NC and CSNK1D -overexpressing HepG2 cells was determined by using CHX (100 μg/ml) at indicated time points. **H** CCK8 assay of HepG2 cells in each group. **I, J** The colony formation of HepG2 cells in each group. **K, L** Sphere formation assays of HepG2 cells in each group. **M, N** Transwell assay of HepG2 cells in each group. **O, P** The wound healing assay of HepG2 cells in each group

The above findings prompted us to further investigate the mechanism that CSNK1D modulated Wnt/β-catenin signaling. To that purpose, we explored the potential direct target or interacting partner of CSNK1D. According to the prediction by HIPPIE and GENEMANIA, CSNK1D might interact with Dishevelled Segment Polarity Proteins (DVLs) (Fig. 6A). Alterations of DVLs have been demonstrated to modulate the Wnt/β-catenin pathway activity in a variety of cancers [31]. Despite the fact that DVL1 and DVL2 exhibited possible significance with HCC in TCGA datasets, no significant changes in DVL1 and DVL2 were observed following CSNK1D knockdown or overexpression (Figure S2). Western blotting revealed that CSNK1D knockdown reduced the expression of DVL3 in MHCC97H cells at the protein level, while overexpression of CSNK1D enhanced the expression of DVL3 in HepG2 cells (Fig. 6B). Based on ROC and Kaplan–Meier analyses in the TCGA, abnormal expression of DVL3 was also associated to the prognosis of HCC patients (Figure S3). We further examined whether CSNK1D regulated the Wnt/β-catenin pathway through DVL3. The stimulation of the Wnt/β-catenin signaling pathway caused by CSNK1D overexpression was abolished by DVL3 deletion (Fig. 6C). For the investigation of molecular mechanism, co-inmunoprecipitation (Co-IP) assay showed that CSNK1D physically interacted with DVL3 in HepG2 cells stably transfected with CSNK1D plasmid (Fig. 6D). Immunofluorescence analysis revealed that CSNK1D and DVL3 were colocalized (Fig. 6E). Overexpression of CSNK1D could significantly alleviated the degradation of DVL3 induced by CHX (Fig. 6F and G). Furthermore, ablation of DVL3 prevented the augmentation of aggressive behaviors generated by CSNK1D overexpression, such as colony formation, proliferation, migration, invasion, and self-renewal of HCC cells (Fig. 6H-P). According to the current findings, CSNK1D may enhance aggressive phenotypes in HCC cells by activating Wnt/β-catenin via interaction with DVL3.

### Overexpression of CSNK1D promotes the tumor growth and metastasis in vivo

**Fig. 7:**
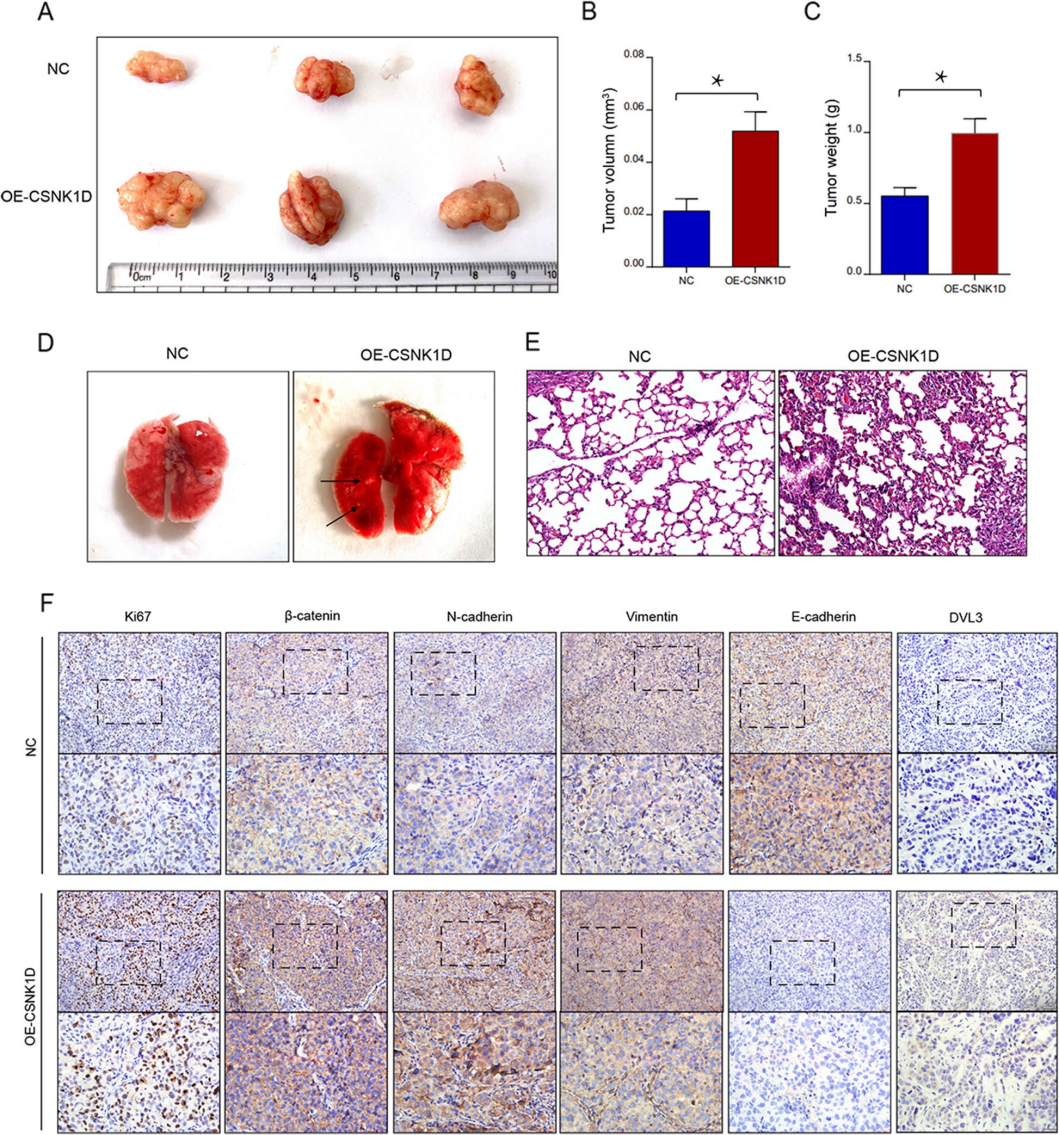
Overexpression of CSNK1D promote the tumor growth and metastasis in vivo. **A** The effect of CSNK1D on tumor growth were evaluated by xenograft tumor in nude mice. **B, C** Volume and weight of xenograft tumors. **D** The effect of CSNK1D on tumor metastasis was observed in lung metastasis model. **E** H&E staining of the lung metastatic nodules. **F** The representative immunohistochemical staining (Ki67, β-catenin, N-cadherin, Vimentin, E-cadherin, DVL3) of the xenograft tumors

To investigate the impact of CSNK1D on HCC growth in vivo, HCC cells with CSNK1D overexpression were injected subcutaneously into nude mice. As illustrated in Fig. 7A-C, the xenograft tumor derived from CSNK1D-overexpressing cells had a larger volume than the NC group. Furthermore, we used a mouse tail vein injection model to test the effect of CSNK1D on HCC cell metastasis. Lung metastasis was detected in the mice of CSNK1D-overexpressing group, while no significant metastasis was found in NC group (Fig. 7D and Fig. 7E). To further validate the effect of CSNK1D on HCC cell growth and EMT phenotypes, we performed IHC analysis in the xenograft tumors. Overexpression of CSNK1D increased the staining intensity of Ki67, β-catenin, N-cadherin and Vimentin, whereas it decreased the expression of E-cadherin (Fig. 7F). These results indicated that CSNK1D facilitated the tumor growth and metastasis in vivo.

## Discussion

The dysregulation of circadian rhythm has been identified as a major contributor to carcinogenesis and tumor growth, according to compelling data [32]. As a result, important circadian rhythm modulators may also play an important role in tumor initiation. As a result, we examined the characteristics of circadian rhythm genes in HCC in depth. The majority of circadian rhythm regulators were found to be overexpressed in HCC tissues in current investigation [29]. We constructed a circadian rhythm genes-based signature upon screening the circadian rhythm genes with prognostic significance, which had excellent performance in predicting the survival of HCC patients. Various signatures for HCC have been established based on the diverse hallmarks of tumor, including metabolism, DNA repair, metastasis, and tumor microenvironment [33]. The current study, for the first time, provided a novel prognostic signature derived from circadian rhythm genes, which may favor distinguishing the poor survival- HCC individuals with omics analysis.

Unlimited proliferation is a hallmark of tumor cell, which is triggered by chronic activation or inhibition of signaling pathways caused by key genes. Previous studies have reported that CSNK1D may have an involvement in the carcinogenesis of a variety of cancers [34–36]. CK1δ, a CSNK1D-coding protein, acts as an oncogenic protein by stabilizing MDM2-p53 and expediting p53 degradation[22, 23]. CSNK1D-coding CK1 Delta has recently been associated to a poor prognosis in HCC [29]. However, the potential functions of CSNK1D and underlying mechanisms remain unknown. In the current investigation, Overexpression of CSNK1D was found in HCC samples from different bioinformatic datasets as well as local HCC samples. Furthermore, increased CSNK1D expression was associated with a decreased survival of HCC patients. In vitro functional validation revealed that CSNK1D overexpression facilitated malignant behaviors including as proliferation, migration, and invasion. In vivo, exogenous CSNK1D has been shown to enhance tumor growth and metastasis. Mechanically, CSNK1D was predicted to modulate Wnt/β-catenin signaling based on bioinformatic study. The Wnt pathway has been implicated in aggressive HCC traits such as proliferation, invasion, chemoresistance, and cancer stem cell features [37]. A recent study demonstrated genetic disruption of the circadian clock hyperactivated Wnt signaling, thereby accelerating Apc-driven tumorigenesis [38]. In the current study, the circadian gene CSNK1D enhanced Wnt/β-catenin activity and downstream genes. However, a Wnt pathway inhibitor was able to greatly reduce the ectopic CSNK1D-induced stimulation of Wnt signaling.

Following that, we uncovered the mechanisms through which CSNK1D modulated Wnt signaling. CSNK1D was discovered to potentially interact with DVLs via protein–protein interaction (PPI) prediction. The DVLs interactome is a well-known regulator of the Wnt pathway, which facilitate inhibition of the destruction complex targeting β-catenin at the cell membrane [39, 40]. According to the experimental validation, knockdown of CSNK1D could only inhibit the expression of DVL3 rather than DVL1 or DVL2. Actually, DVL3 has been implicated in multiple malignant behaviors, including cell proliferation, metastasis and cancer stem cell properties [41, 42]. In this study, the abnormal expression of DVL3 in HCC and its prognostic significance were validated by bioinformatic analyses. Co-IP assay indicated that CSNK1D could physically interact with DVL3 protein. In addition, such interaction could protect DVL3 against CHX-induced degradation. Thus, it is proposed that CSNK1D could regulate Wnt signaling by interacting with DVL3. Furthermore, silencing DVL3 inhibited the enhancement of HCC cell growth, colony formation, as well as the stimulation of migration elicited by ectopic CSNK1D, implying that CSNK1D could enable aggressive behaviors by activating the DVL3/Wnt axis.

In this study, the circadian rhythm gene CSNK1D expression level was significantly correlated with clinical parameters of HCC patients, including tumor stage, differentiation, and survival status. Though it showed potential as a candidate biomarker, the overall predictive capacity may be significantly improved by combing some widely acknowledged markers for joint analysis. In addition, our findings provide evidence that CSNK1D has an oncogenic effect on HCC progression, particularly in terms of favoring malignant phenotypes. Indeed, IC261, a CK1δ and CK1ε inhibitor, inhibited HCC cell proliferation and promoted cell death in a time and dose-dependent way [24]. However, it currently lacks an agent specifically targeting CSNK1D or CK1δ. Despite the positive findings, there are still certain restrictions. The present cohort used to validate the signature and CSNK1D's predictive usefulness is limited, and these markers should be examined in larger multi-center cohorts. Moreover, the correlation of CSNK1D-mediated circadian rhythm with HCC progression remains unclear, which needs more experiments to investigate and identify the underlying mechanisms.

## Conclusion

In conclusion, a robust signature was identified based on circadian genes for HCC patients. Furthermore, the circadian gene CSNK1D was significantly upregulated in HCC and enhanced malignant behaviors via Wnt signaling pathway by stabilizing DVL3. It suggests that CSNK1D is a potential marker and therapeutic target of HCC, which urges more studies for further investigation.

## Supplementary Information

**Additional file 1:** **Figure S1. **Kaplan-Meier analysis of patients at different time point.The overall survival curves and recurrence free survival curves at 3, 5, or 10-year of HCC patients with high or low CSNK1D expression in of TCGA LIHC cohort. **FigureS2. **The clinical implications and correlations with CSNK1D.** (A) **The expression of DVL1 and DVL2 in HCC and normal liver tissues in TCGA. **(B) **The ROC for HCC patients in TCGA dataset. **(C) **The effects of CNSK1D knockdown or overexpression on DVL1/2 expression. ***P< 0.001. **Figure S3. **The clinical implications of DVL3 for HCC patients.** (A)** The expression of DVL3 in HCC and normal liver tissues in TCGA.** (B)** The ROC curves for HCC patients regarding the expression of DVL3 expression.** (C) **The Kaplan-Meier curvesfor HCC patients regarding the expression of DVL3 expression. ***P< 0.001.

## Data Availability

All data generated or analyzed during this study are included in this published article. All of the data and material in this study are available when requested.

## Abbreviations

HCC: Hepatocellular carcinoma
TCGA-LIHC: The cancer genome atlas liver hepatocellular carcinoma
CSNK1D: Casein Kinase 1 Delta
IHC: Immunohistochemistry
WB: Western blotting
qRT-PCR: Quantitative real-time PCR
CCK8: Cell counting kit-8
LASSO: Least absolute shrinkage and selection operator
EMT: Epithelial-mesenchymal transformation
DVL3: Dishevelled Segment Polarity Protein 3
CK1: Casein kinase 1
UALCAN: Update to the integrated cancer data analysis platform
HR: Hazard ratio
ROC: Receiver operating characteristic
GSE: Gene Expression Omnibus Series
NCBI: National Center for Biotechnology Information
GEO: Gene Expression Omnibus
GSEA: Gene set enrichment analysis
FDR: False discovery rate
ShRNA: Short hairpin RNA
siRNA: Small interfering RNA
PFA: Paraformaldehyde
H&E: Hematoxylin-eosin staining
BSA: Bovine serum albumin
TBS: Tris buffered saline
DAB: Diaminobenzidine
DAPI: 4’,6-Diamidino-2’-phenylindole
RIPA: Radioimmunoprecipitation assay buffer
SDS-PAGE: Sodium dodecyl sulfate polyacrylamide gel electrophoresis
PVDF: Polyvinylidene fluoride
SDS: Sodium dodecyl sulfate
IB: Immunoblotting
GAPDH: Glyceraldehyde phosphate dehydrogenase
Ct: Cycle threshold
OS: Overall survival
RFS: Recurrence free survival
TNM: Tumor node metastasis
EMT: Epithelial-mesenchymal transition
DEGs: Differential expressed genes
CSC: Cancer stem cell
DVLs: Dishevelled segment polarity proteins
Co-IP: Co-inmunoprecipitation
CHX: Cycloheximide
PPI: Protein-protein interaction
