# Supplementary material for: Circadian gene CSNK1D promoted the progression of hepatocellular carcinoma by activating Wnt/β-catenin pathway via stabilizing Dishevelled Segment Polarity Protein 3
Source: Biol Proced Online. 2022 Dec 2;24:21. doi: 10.1186/s12575-022-00183-x (PMC9717411; doi:10.1186/s12575-022-00183-x)
Supplement: Supplementary file 1 — Additional file 1: Figure S1. Kaplan-Meier analysis of patients at different time point.The overall survival curves and recurrence free survival curves at 3, 5, or 10-year of HCC patients with high or low CSNK1D expression in of TCGA LIHC cohort. FigureS2. The clinical implications and correlations with CSNK1D. (A) The expression of DVL1 and DVL2 in HCC and normal liver tissues in TCGA. (B) The ROC for HCC patients in TCGA dataset. (C) The effects of CNSK1D knockdown or overexpression on DVL1/2 expression. ***P< 0.001. Figure S3. The clinical implications of DVL3 for HCC patients. (A) The expression of DVL3 in HCC and normal liver tissues in TCGA. (B) The ROC curves for HCC patients regarding the expression of DVL3 expression. (C) The Kaplan-Meier curvesfor HCC patients regarding the expression of DVL3 expression. ***P< 0.001. [file 12575_2022_183_MOESM1_ESM.docx]

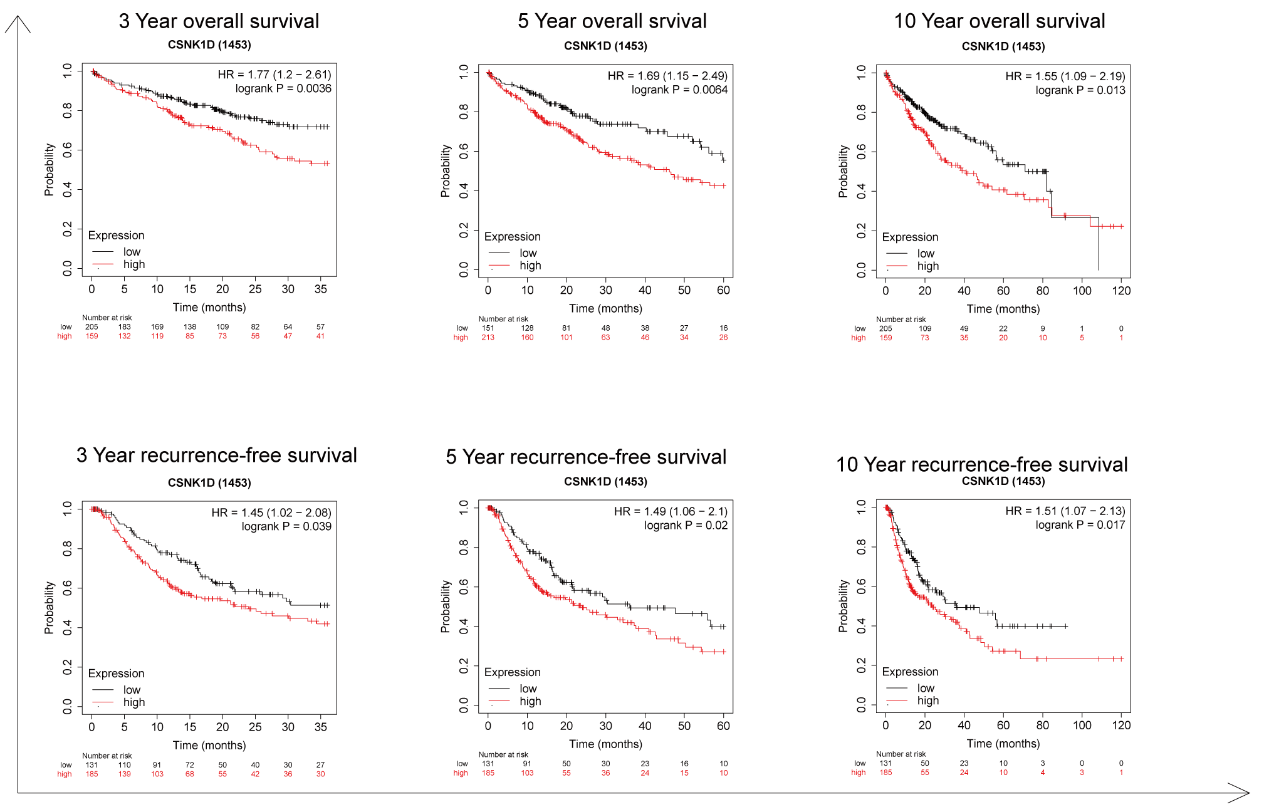


**Figure S1. Kaplan-Meier analysis of patients at different time point.** The overall survival curves and recurrence free survival curves at 3, 5, or 10-year of HCC patients with high or low CSNK1D expression in of TCGA LIHC cohort.

**
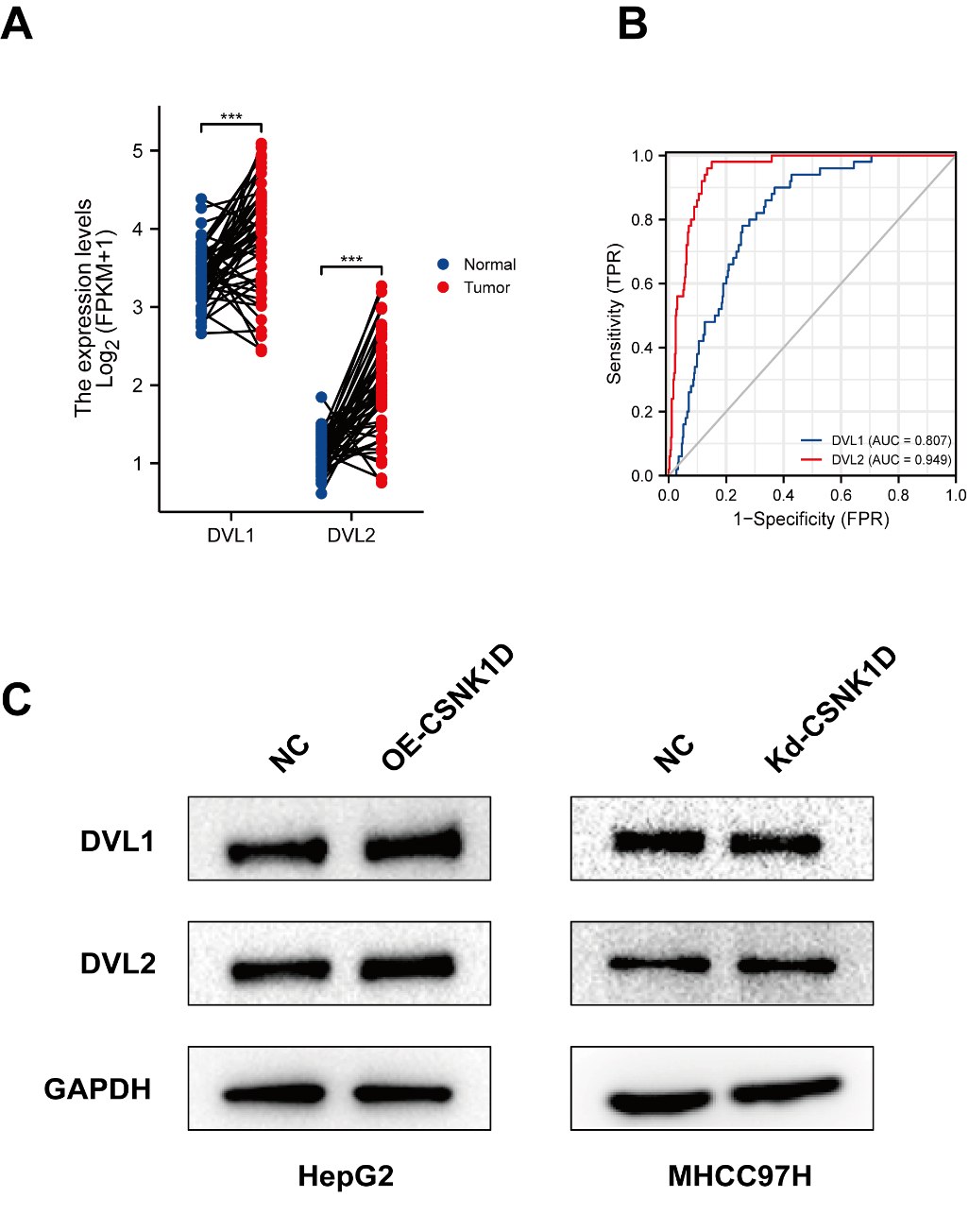
**

**Figure S2. The clinical implications and correlations with CSNK1D. (A)** The expression of DVL1 and DVL2 in HCC and normal liver tissues in TCGA. **(B)** The ROC for HCC patients in TCGA dataset. **(C)** The effects of CNSK1D knockdown or overexpression on DVL1/2 expression. ***P < 0.001.

**Figure S3**

**
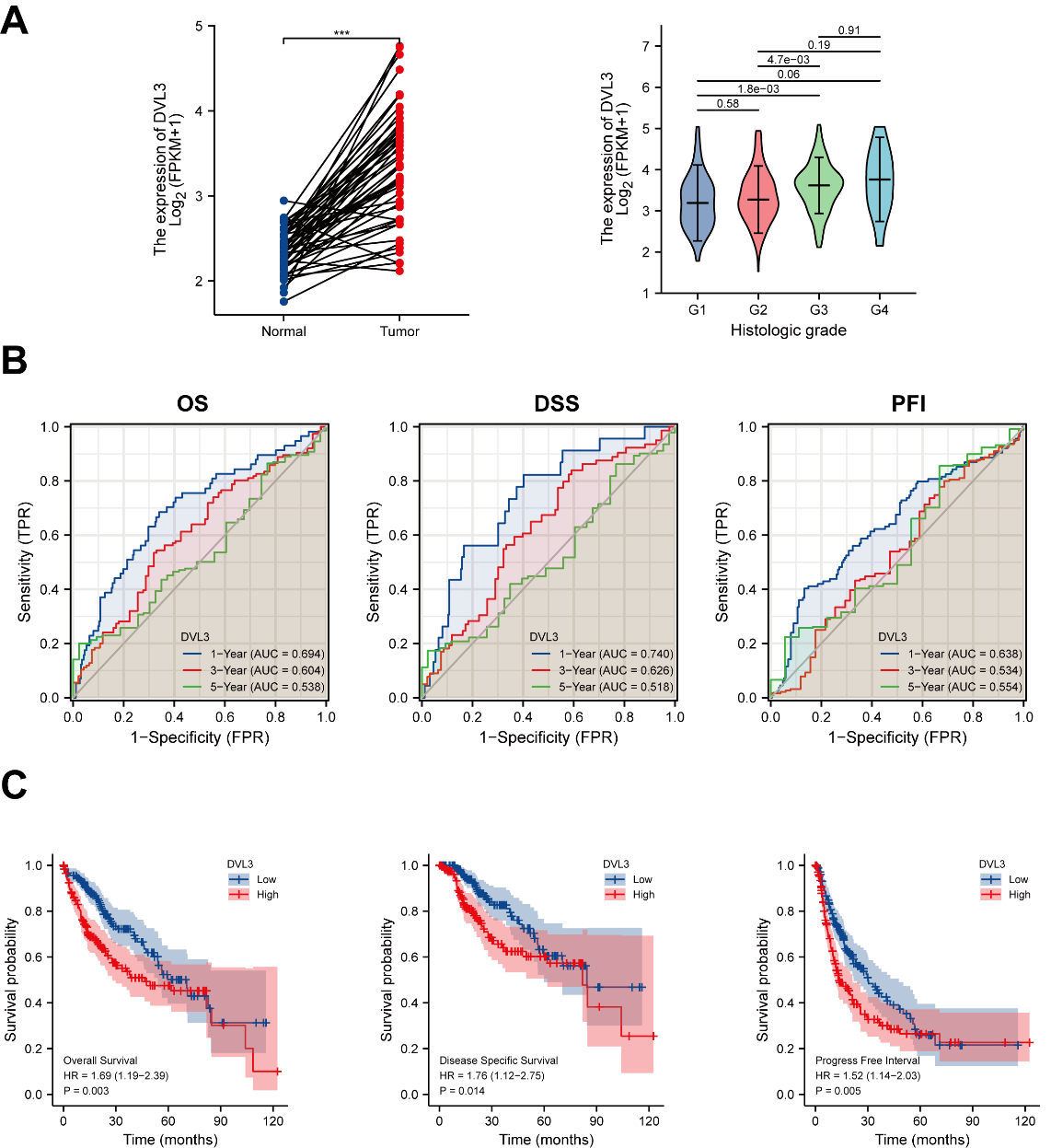
.**

**Figure S3. The clinical implications of DVL3 for HCC patients. (A)** The expression of DVL3 in HCC and normal liver tissues in TCGA. **(B)** The ROC curves for HCC patients regarding the expression of DVL3 expression. **(C)** The Kaplan-Meier curves for HCC patients regarding the expression of DVL3 expression. ***P < 0.001.
